# Editorial: Innovative approaches in psychosocial and mental health

**DOI:** 10.3389/fpubh.2026.1907588

**Published:** 2026-06-26

**Authors:** Carina Florin, Elke Humer, Harald Baumeister, Thomas Probst

**Affiliations:** 1 Division of Psychotherapy, Department of Psychology, University of Salzburg, Salzburg, Austria; 2 Department for Psychosomatic Medicine and Psychotherapy, University for Continuing Education Krems, Krems, Austria; 3 Department of Clinical Psychology and Psychotherapy, Institute of Psychology and Education, Ulm University, Ulm, Germany; 4 German Center for Mental Health (DZPG), Partner Site Mannheim-Heidelberg-Ulm, Ulm, Germany; 5 German Center for Child and Adolescent Health (DZKJ), Partner Site Ulm, Ulm, Germany

**Keywords:** biologically enhanced psychotherapy, continuous support, psychosocial interventions, telehealth and digital solutions, virtual reality therapy

## 1 Introduction

Globally, rising prevalence and complexity of mental health issues and psychosocial needs demand innovative, evidence-based approaches (1–3). Building on this, a recent study by Patel et al. (4) argues that mental health care is overly constrained by diagnostic categories and should transition to models that stratify care by severity and staging while systematically integrating psychosocial interventions. Accordingly, there is a compelling need to thoroughly design and evaluate cutting-edge psychosocial interventions that integrate technology, biologically augmented psychotherapy, and continuous support to address complex mental health challenges and improve care (5, 6).

The 24 submissions to this topic accepted for publication can, for the purpose of synthesis, be organized into five categories.

### 1.1 Internet- and mobile-based interventions (IMI)

This section depicts studies on digital and remotely delivered mental health interventions across various disorders and settings, highlighting usability, effectiveness, implementation, and emerging AI-enabled approaches.

To begin with, mobile applications for eating disorders offer flexible, cost-effective delivery of evidence-based interventions but face challenges with engagement and adherence. Albano et al. delivered the INTERconNEcT-eating disorders guided self-help program in the aChiral Content app for usability and user-experience evaluation. They found that it demonstrated satisfactory usability and acceptability among individuals with eating disorders, and that user-centered design with peer involvement appeared to enhance engagement.

Moreover, the study of Al-Mahrouqi et al. examined Omani psychologists' perceptions of telephone-based psychotherapy with respect to service quality, accessibility, and acceptability. The psychologists viewed phone consultations as a valuable adjunct that improves access and reduces stigma, while emphasizing that high-risk or acutely unwell clients require in-person evaluation for safety and efficacy.

Next, physicians and trainees experience elevated burnout, anxiety, and depression, yet often avoid support due to stigma, time constraints, and limited access. A cognitive-behavioral-based supportive SMS intervention study by Obeng Nkrumah et al. showed reductions in emotional exhaustion and anxiety, with high user satisfaction, accessibility, and low cost. This indicates promise as a scalable complement to traditional mental health services for healthcare professionals.

In addition, the meta-analysis by Fialho et al. evaluated the effectiveness of digital interventions for psychosis and examined dose characteristics in relation to outcomes. It found preliminary evidence of effectiveness, but no significant dose–response relationship, indicating a need for further research.

The study of Zarski et al. developed and tested the feasibility of an AI-based Just-In-Time-Adaptive-Intervention (DyAI-JITAI) app designed to bridge the intention–behavior gap by supporting daily cognitive behavioral skill use with optional buddy motivation, thereby informing user-centered design, procedures, and safety considerations for a future large-scale randomized controlled efficacy trial.

Zeng et al. designed a study to investigate the effectiveness of therapist-guided internet-based mindfulness-based cognitive therapy for insomnia. After effectiveness is proven, this study could provide evidence for clinical practitioners and therapists to consider this new psychotherapeutic option for patients.

Moreover, Thaysen-Petersen et al. explored the efficacy of conventional and technology assisted cue exposure therapy for treating substance use disorders in a qualitative systematic review. The study showed that technology-assisted cue exposure therapy, particularly virtual reality, more often showed significant benefits, highlighting the need for high-quality trials to enable firmer conclusions.

It is worth noting that European health systems are struggling to recruit and retain healthcare workers, as mental health problems are a major driver of staff turnover. Yet, despite the availability of effective eHealth tools, few have been tested under real-world conditions. The study by Garcia-Vazquez et al. evaluating the “Doing What Matters in Times of Stress” intervention among Madrid primary care healthcare workers found good implementation, provided evidence supporting hybrid/remote delivery and highlighted access barriers.

### 1.2 Big data and data from social media in mental health

The next two contributions leverage social media analytics and advanced language models to describe public discourse around psychotherapy and the construction of meaning in everyday contexts.

Social media platforms provide an opportunity to examine the perception of psychotherapy. Accordingly, Goena et al. analyzed how psychotherapy is discussed on X over the last 15 years, including Spanish and English tweets. The authors noticed that linguistic differences shape the discourse on social media, highlighting the need for culturally tailored communication.

Li et al. explored the meaning of life from social network content in the sleep scenario through deep learning models and semantic dependency graph algorithms to find associated factors and construct subnetworks for further analysis of factors, interrelations and differences.

### 1.3 Novel therapeutic modalities

The following contributions chart key advances and challenges at the intersection of technology, psychotherapy, and mental health research and practice.

A case series by Wollweber et al. focused on AI-personalized visualization of the safe place in virtual reality vs. imagination in terms of stress, burnout, and relaxation in psychotherapists, showing that brief imaginations and VR interventions enhance relaxation in psychotherapists.

Turning to implementation barriers of virtual reality, Felnhofer et al. found that lack of training, financial constraints, and limited motivation are key barriers to adopting virtual reality applications for patient use in Austrian clinical psychologists and psychotherapists.

From individual interventions to the wider field, the systematic review of the integration and development trends between arts and psychotherapy by Xie and Sun revealed structural problems, insufficient connections between technical applications and practice guidelines as well as inadequate coverage of certain groups.

Methodically, the study of Shen et al. explored films portraying autistic individuals revealing systematic rhythmic patterns and distinct differences in shot length, editing, camera movement, and composition, offering an objective basis for analyzing emotional portrayal. These insights support practical applications in training and intervention design and enhance public understanding of autism spectrum disorder.

Pointing to emerging therapeutic opportunities, the study protocol by Peter et al. focuses on the complex interplay of smoking and mental health and their connection to metabolism and the gut microbiome, thereby addressing novel therapeutic modalities.

### 1.4 Psychophysiological, psychosocial, and symptom dynamics

This section advances understanding of mechanisms and patterns across psychophysiological, psychosocial, behavioral, and symptom dynamics.

Among island-based firefighters sleep disturbances, fatigue, and psychological distress are common. Liu et al. mapped relationships among these factors and psychological resilience. Depression and daytime dysfunction emerged as key bridging nodes between sleep and affective processes. Fatigue appeared to influence distress via sleep pathways. Tenacity showed an upstream protective role, while sleep status or shift work could reorganize network structure without changing overall connectivity.

Furthermore, Song et al. examined how nap duration and nighttime sleep relate to depression among Chinese residents to inform evidence-based sleep recommendations for prevention. Nighttime sleep of seven to 9 h and naps of 30–90 min were linked to fewer depressive symptoms, with effects varying by health status and age, underscoring the need for tailored guidance.

Turning to the question of how sports participation influences the fertility intentions of married young adults in China, Lin et al. highlighted the roles of both marital and family subsystems in shaping decisions. The findings suggest that policies encouraging sports participation alongside family-centered support may effectively enhance fertility intentions in this population.

Moreover, Skurvydas et al. compared physical activity, sleep, body-mass-index, subjective health, stress, depression, impulsivity, and emotional intelligence across the Baltic countries to identify key health determinants. Perceived health was inversely associated with age, stress, depression, and body-mass-index, and positively associated with vigorous physical activity as well as emotional intelligence.

### 1.5 Mental health systems and community-based care

This section highlights evidence on community- and system-level mental health approaches.

A community case study by Wilson et al. presented a Tiny Home Village model to expand affordable housing for people with severe mental illness, a group at increased risk of housing insecurity due to low incomes. It points out how cross-sector collaboration can address entrenched public health challenges and offers a scalable blueprint for increasing affordable housing supply for this vulnerable population.

Thermaenius et al. evaluated the “Family Talk Intervention” implementation over time in palliative care settings from hospital social workers' perspectives. Hospital social workers rated the implementation as largely positive and useful in routine practice, though lower scores for managerial support and resources highlight contextual gaps.

The study by Bogatzki et al. evaluated a pilot project called “Coordinated Psychotherapeutic Treatment with the Involvement of Peer Support Workers” integrating supervised autonomous peer support workers to help refugees with mental disorders navigate Germany's healthcare system. Peer support workers addressed key utilization barriers, reported both challenges and benefits, and underscored the importance of training and regular supervision, indicating that peer-based approaches can enhance equitable access to indicated psychotherapy for this underserved population.

Moreover, Seah et al. elucidated providers' perspectives on barriers to mental healthcare in Singapore, identifying systemic under-resourcing and poor cross-sector coordination as key drivers of care gaps. Recommendations include expanding insurance coverage, strengthening private-sector regulation, reforming reimbursement to support integrated care, streamlining referrals and data sharing and advancing destigmatization and community-based services to improve timely and equitable access.

Next, the study of Elkin et al., conducted at a private university in Istanbul, presented an assessment of university students' mental health literacy and happiness, examining their association. Findings suggest that higher mental health literacy and access to mental health resources are linked to greater happiness, indicating that expanding mental health literacy and resource availability may enhance wellbeing.

## 2 Conclusion

The Research Topic “*Innovative Approaches in Psychosocial and Mental Health*” brings together a collection of systematic reviews, study protocols, original research, and case studies that showcases innovative approaches with clear implications for mental health strategies. Together, these contributions strengthen conceptual and practical understanding, identifying scalable strategies to improve care and access and demonstrating the importance of research in this field. This Research Topic points out the need for continued support and thorough evaluation of novel interventions to advance mental health research and clinical practice.
